# Qualitative monitoring of SARS-CoV-2 mRNA vaccination in humans using droplet microfluidics

**DOI:** 10.1172/jci.insight.166602

**Published:** 2023-07-10

**Authors:** Matteo Broketa, Aurélien Sokal, Michael Mor, Pablo Canales-Herrerias, Angga Perima, Annalisa Meola, Ignacio Fernández, Bruno Iannascoli, Guilhem Chenon, Alexis Vandenberghe, Laetitia Languille, Marc Michel, Bertrand Godeau, Sébastien Gallien, Giovanna Melica, Marija Backovic, Felix A. Rey, Jean Baudry, Natalia T. Freund, Matthieu Mahévas, Pierre Bruhns

**Affiliations:** 1 Institut Pasteur, Université Paris Cité, Inserm UMR1222, Antibodies in Therapy and Pathology, 75015 Paris, France.; 2 Diaccurate SA, Paris, France.; 3 Sorbonne Université, Collège Doctoral, F-75005 Paris, France.; 4 Institut Necker Enfants Malades (INEM), INSERM U1151/CNRS UMS 8253, Université de Paris, Paris, France.; 5 Department of Clinical Microbiology and Immunology, Sackler Faculty of Medicine, Tel Aviv University, Tel Aviv, Israel.; 6 Institut Pasteur, Université Paris Cité, CNRS UMR3569, Structural Virology, 75015 Paris, France.; 7 Laboratoire Colloïdes et Matériaux Divisés (LCMD), ESPCI Paris, PSL Research University, CNRS UMR8231 Chimie Biologie Innovation, Paris, France.; 8 Service de Médecine Interne, Centre Hospitalier Universitaire Henri-Mondor, Assistance Publique-Hôpitaux de Paris (AP-HP), Université Paris-Est Créteil (UPEC), Créteil, France.; 9 INSERM U955, Équipe 2. Institut Mondor de Recherche Biomédicale (IMRB), Université Paris-Est Créteil (UPEC), Créteil, France.; 10 Service de Maladies Infectieuses, Centre Hospitalier Universitaire Henri-Mondor, Assistance Publique-Hôpitaux de Paris (AP-HP), Université Paris-Est Créteil (UPEC), Créteil, France.; 11 Paris Est Créteil University UPEC, Assistance Publique-Hôpitaux de Paris (AP-HP), Henri Mondor Hospital, Fédération Hospitalo-Universitaire TRUE InnovaTive theRapy for immUne disordErs, Créteil, France.

**Keywords:** Immunology, Vaccines, Adaptive immunity, Immunoglobulins

## Abstract

SARS-CoV-2 mRNA vaccination generates protective B cell responses targeting the SARS-CoV-2 spike glycoprotein. Whereas anti-spike memory B cell responses are long lasting, the anti-spike humoral antibody response progressively wanes, making booster vaccinations necessary for maintaining protective immunity. Here, we qualitatively investigated the plasmablast responses by measuring from single cells within hours of sampling the affinity of their secreted antibody for the SARS-CoV-2 spike receptor binding domain (RBD) in cohorts of BNT162b2-vaccinated naive and COVID-19–recovered individuals. Using a droplet microfluidic and imaging approach, we analyzed more than 4,000 single IgG-secreting cells, revealing high interindividual variability in affinity for RBD, with variations over 4 logs. High-affinity plasmablasts were induced by BNT162b2 vaccination against Hu-1 and Omicron RBD but disappeared quickly thereafter, whereas low-affinity plasmablasts represented more than 65% of the plasmablast response at all time points. Our droplet-based method thus proves efficient at fast and qualitative immune monitoring and should be helpful for optimization of vaccination protocols.

## Graphical abstract

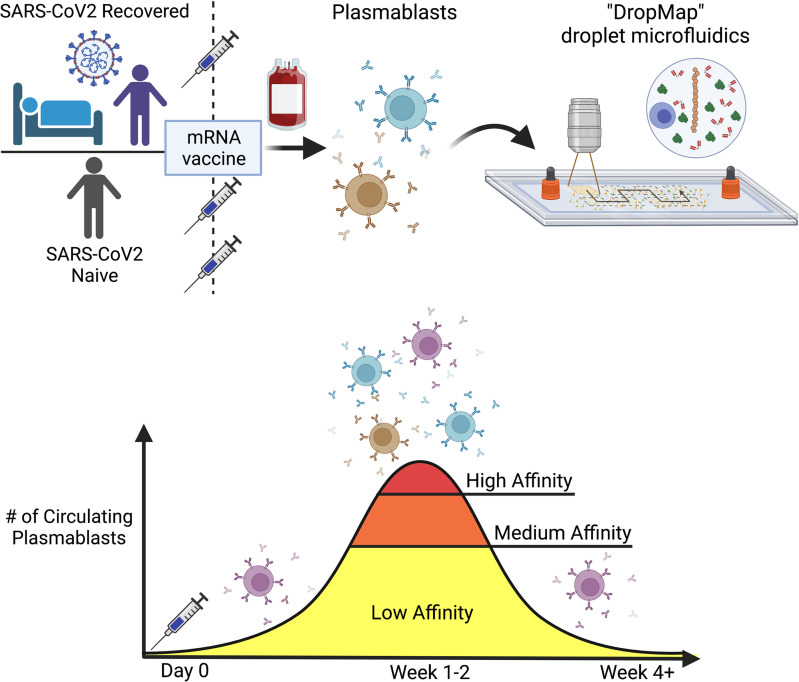

## Introduction

Since the SARS-CoV-2 pandemic began in early 2020, close to 760 million cases of infection and over 6.8 million deaths have been reported by the WHO. The emergency use authorization by drug administration agencies worldwide allowed anti–SARS-CoV-2 vaccination to start by the end of 2020, with over 5.5 billion people receiving at least 1 dose and more than 5.1 billion fully vaccinated (at least 2 doses) as of April, 2023. This astounding global vaccination effort has been kick-started by mRNA-based vaccines encoding a stabilized version of the trimeric spike protein of SARS-CoV-2, developed by either Moderna or Pfizer-BioNTech. Both vaccines had indeed demonstrated rapid and strong immune responses and efficacy in phase III studies, eliciting effective B cell–dependent humoral immunity, with high titers of neutralizing antibodies, which are key to prevention of severe COVID-19 and mortality (1–7). Yet, the neutralizing antibody titers in sera from vaccinees decreased rapidly for the first 3 months, with a relatively slow decrease thereafter (8, 9).

An additional challenge is the rapid emergence of SARS-CoV-2 variants, particularly with mutations in the receptor-binding domain (RBD) of the spike protein, raising concerns that the immunity raised against the initial SARS-CoV-2 strain Wuhan-Hu-1 (termed Hu-1 herein), either following natural infection or following vaccination, would not be protective against SARS-CoV-2 variants of concern (VOCs) (10, 11). Whereas most VOCs (B.1.1.7 [Alpha], B.1.351 [Beta], P.1 [Gamma], B1.617.2 [Delta]) harbor relatively few (8–12) amino acid mutations in their spike protein as compared with the original Hu-1 strain, the Omicron BA.1, BA.2, BA.3, and BA.4/BA.5 variants harbor 24–34 mutations, making immune escape by these latter variants highly probable (12). Most clinically approved monoclonal antibodies (mAbs) indeed fail to protect from these newest variants (13, 14). This continuous emergence of novel viral variants isolated from patients drives a continuous vaccination effort to induce long-term immune responses with the hope to cover all present and future viral variants.

The main efforts to characterize the patient’s immune response to natural infection and/or vaccination focused on serological profiling of the circulating neutralizing antibody responses, and on spike- or RBD-specific memory B cells (MBCs), from which a selection of antibodies was expressed and assayed in vitro for neutralization potency and binding affinity (15, 16). While convergent results following natural infection and/or vaccination report a fast decrease in serum neutralization over the first 3 months, the MBC response over time remains relatively stable against the original Hu-1 strain as well as most VOCs, with antibodies expressed from these repertoires exhibiting a wide range of 0.2–2,000 nM binding affinity against Hu-1 spike/RBD (3, 7, 15, 17). Moreover, the cross-reactivity of anti–Hu-1 antibody and MBC responses with Omicron was reported to be approximately 30% after 2 vaccine doses (18, 19) and up to 50% after 3 doses (16).

Rare studies have included the analysis of spike-specific antibody-secreting cells (ASCs), i.e., plasmablasts and plasma cells. Two investigations published by the Ellebedy lab analyzed bone marrow cells and fine-needle aspirates of lymph nodes (9, 20). These studies described expression of antibodies from bone marrow, lymph node plasma cells, and circulating plasmablasts, with up to 1,500 mAbs recombinantly expressed for a single study (20). This impressive work nevertheless required single-cell sorting, antibody variable region (VH-VL) gene sequencing, repertoire analyses, cloning/synthesis, recombinant expression, and finally, affinity measurements. Therefore, while being extremely informative, such an approach entails a large workload, cost, and time investments, and thus the total number of antibodies that can be eventually analyzed is limited.

The ability to measure large numbers of spike-specific antibody binding affinity directly can shed light on the immune response following infection or vaccination in a rapid fashion that will help evaluate vaccination protocols and public health measures. We describe here an adaptation for such human studies of a droplet-based microfluidic technology (DropMap) with same-day results obtained at highly reduced workload and costs (21). DropMap combines the immobilization within an observation chamber of approximately 100,000 picoliter-size droplets containing nonsorted single cells with an ultrasensitive fluorescent bioassay. DropMap was reported for mouse studies to qualitatively follow immune responses to protein immunization (22), bacteria (23), viruses (24), and abnormal IgG secretion (25), and for clinical studies to measure single-cell cytokine secretion from sepsis patients (26), cytotoxicity from single human NK cells (27), and affinity and secretion rate of IgGs secreted by autoreactive IgG-secreting cells from blood, spleen, and bone marrow of patients with immune thrombocytopenia (28). Using this massively parallel kinetic analyses of single ASCs, we evaluated the initial circulating ASC responses in cohorts of individuals vaccinated with BNT162b2 (Pfizer-BioNTech). Two cohorts were included, one of individuals with no prior SARS-CoV-2 infection (COVID-19–naive), and one of individuals who had recovered from a SARS-CoV-2 infection 1 year before (COVID-19–revovered). We followed these cohorts over time, after 1 or 2 doses of SARS-CoV-2 mRNA vaccine and analyzed their ASC response against Hu-1 RBD, and for selected individuals also for BA.1 RBD.

## Results

### Single-cell bioassay allows phenotypic characterization of RBD-specific ASCs.

For this study, we adapted a single-cell bioassay in microfluidic droplets termed DropMap that we have described previously (22, 26, 28). DropMap allows for a direct sorting-free assessment of the antibody-based cellular immune response, while characterizing the secretion rate, specificity, and affinity for SARS-CoV-2 Hu-1 RBD of human IgG secreted by circulating plasmablasts and plasma cells, which here we collectively term IgG-secreting cells (IgG-SCs), allowing for analysis of 10,000–20,000 cells/hour.

**Figure 1:**
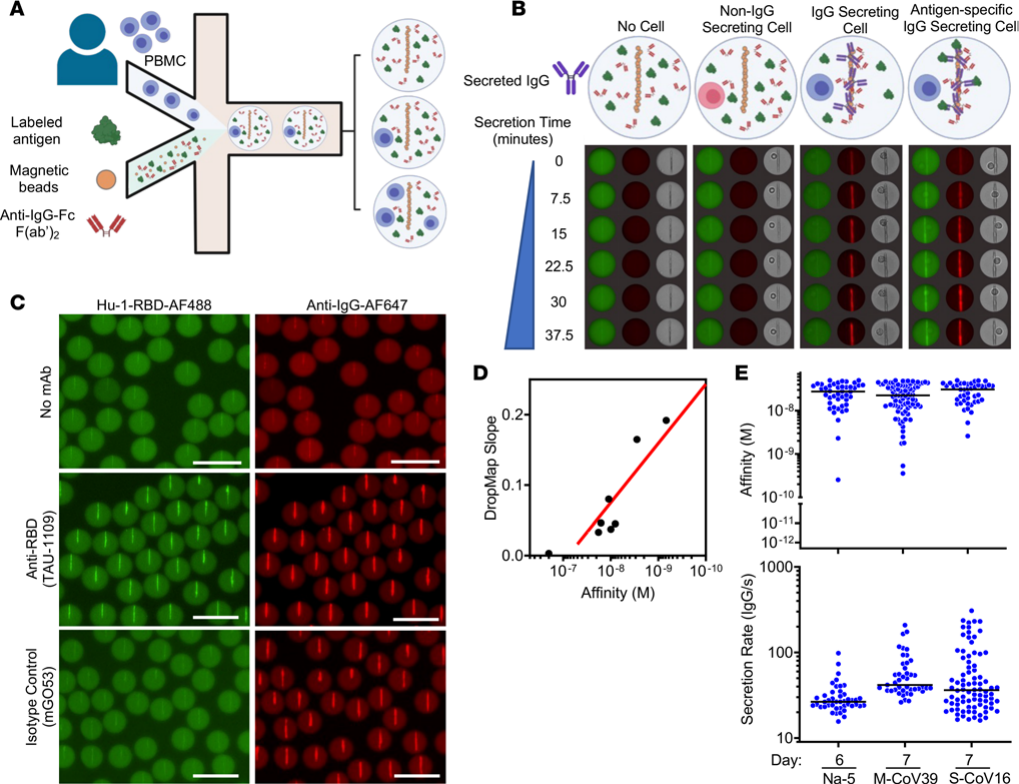
DropMap technique for the detection of anti–Hu-1 IgG–secreting cells. (**A**) Schematic showing the inputs and outputs of microfluidic encapsulation. PBMCs (top) are flowed in parallel with bioassay components (RBD–Alexa Fluor 488, magnetic beads coated with anti–κ light chain nanobody (VHH), and anti-IgG F(ab′)_2_–Alexa Fluor 647) on the droplet nozzle. Oil flow closes the collected aqueous phases (cell component and bioassay component) into a water-in-oil droplet. The column of droplets depicts the potential outcomes of cell encapsulation. (**B**) Schematic showing the composition of the DropMap assay and potential changes to the assay as it progresses. In the absence of a cell (outer left) or with a non–IgG-SC (inner left), there is no change in RBD (green) or anti–IgG F(ab′)_2_ (red) fluorescence distributions over time. An IgG-SC (inner right) in the droplet causes anti–IgG F(ab′)_2_ (red) fluorescence to relocate to the beadline over time. An RBD-specific IgG-SC (outer right) causes both RBD (green) and anti–IgG F(ab′)_2_ (red) fluorescence to relocate to the beadline. (**C**) Example of droplets containing no mAb (top), 5 nM TAU-1109 anti-RBD mAb (center), or 5 nM isotype control IgG (clone mGO53, bottom), showing fluorescence relocation of Hu-1 RBD–Alexa Fluor 488 (green, left) and anti–IgG F(ab′)_2_–Alexa Fluor 647 (red, right). Scale bars: 50 μm. (**D**) Hu-1 RBD affinity reference curve calibrated with anti-RBD mAbs with known *K_D_* and DropMap slope determined from methods exampled in (Supplemental Figure 1B). Each dot represents the values for 1 mAb or isotype control. (**E**) Representative examples of anti-RBD antibody affinity (top) and IgG secretion (bottom) by circulating, single IgG-SCs (*n* = 171) from indicated individuals; each dot represents 1 IgG-SC; black bars indicate median values.

Human peripheral blood mononuclear cells (PBMCs) of volunteers were coencapsulated in droplets along with paramagnetic nanoparticles and fluorescent bioassay reagents that were immobilized within an observation chamber and imaged over 1 hour by time-lapse fluorescence imaging (Figure 1A and Supplemental Figure 1A; supplemental material available online with this article; https://doi.org/10.1172/jci.insight.166602DS1). Including paramagnetic beads in the droplets allows the use of a magnetic field to align them and make a clearly distinguishable pattern (termed the “beadline”) by microscopy. The beads were coated with an IgG capture reagent, and the droplets contained a fluorescently labeled IgG detection reagent and fluorescently labeled monomeric RBD, leading to a fluorescent beadline when IgG is secreted within the droplet. The beadline thus serves as a physical surface for a double-fluorescent sandwich immunoassay, revealing IgG secretion from the cell and specificity of that IgG for the antigen (i.e., Hu-1 RBD; Figure 1, B and C). Image analyses consisted of 5 stages: (i) identifying each droplet and its spatial coordinates within the DropMap chamber; (ii) identifying the beadline within each droplet; (iii) selecting droplets containing a single cell; (iv) extracting for each time point the fluorescence signals of the beadline, and of the entire droplet without the beadline; and (v) calculating the fluorescence accumulation on the beadline over time. Each droplet is assessed individually, both computationally and visually, to ensure proper assay formation and data fidelity. The time-resolved fluorescence signals allow for the estimation of IgG secretion rates and affinity for RBD of the secreted IgG by using calibration curves generated with a monoclonal anti-RBD IgG with known affinity (*K_D_*) encapsulated at various concentrations (Supplemental Figure 1B). The resulting anti–Hu-1 RBD reference curve, generated using 7 different anti-RBD IgG mAbs of various affinities (29), allows for measurements with certainty over 2 logs of affinities, i.e., 2 × 10^–10^ ≤ *K_D_* ≤ 5 × 10^–8^ M (Figure 1D). Therefore herein, IgG interacting with RBD at a calculated *K_D_* below 5 × 10^–8^ M is considered binding, i.e., anti-RBD IgG antibodies, and the cell secreting such IgGs is termed RBD-specific IgG-SCs.

Selected individuals were part of 2 longitudinal cohorts: a first cohort of recovered COVID-19 patients (4 severe [S-CoV] and 14 mild [M-CoV]), which is part of the MEMO-COV-2 cohort (7) followed for 1 year after initial infection prior to vaccination; and a second cohort of 11 COVID-19–naive healthcare workers, with no clinical history of COVID-19 and no serological evidence of previous SARS-CoV-2 infection (verified by the absence of anti–SARS-CoV-2 nucleocapsid antibodies), vaccinated as part of the French vaccination program. All individuals received the BNT162b2 vaccine and were sampled for circulating IgG-SC analyses after prime and/or boost (17). At the time of this clinical study, COVID-19–naive individuals could receive a primary vaccination and a booster vaccination, whereas COVID-19–recovered individuals were considered already primed by the infection, and therefore were given only 1 vaccine dose. As an example, blood was obtained from 1 COVID-19–naive donor (donor Na-5; 33 y/o, male) who was sampled 6 days after booster vaccination, from 1 mild COVID-19–recovered patient (patient M-CoV39; 34 y/o, male) and 1 severe COVID-19–recovered patient (patient S-CoV16; 61 y/o, male) who were sampled 7 days after vaccination (Figure 1E), with detectable Hu-1 RBD–specific IgG-SCs for all 3 individuals. These cells displayed a large range of secretion rates of IgG (~15–300 molecules per second [IgG/s]), with median values of approximately 30, 60, and 60 IgG/s in these 3 individuals, respectively.

### Anti–Hu-1 RBD IgG-SC populations following vaccination in naive or COVID-19–recovered individuals.

**Figure 2:**
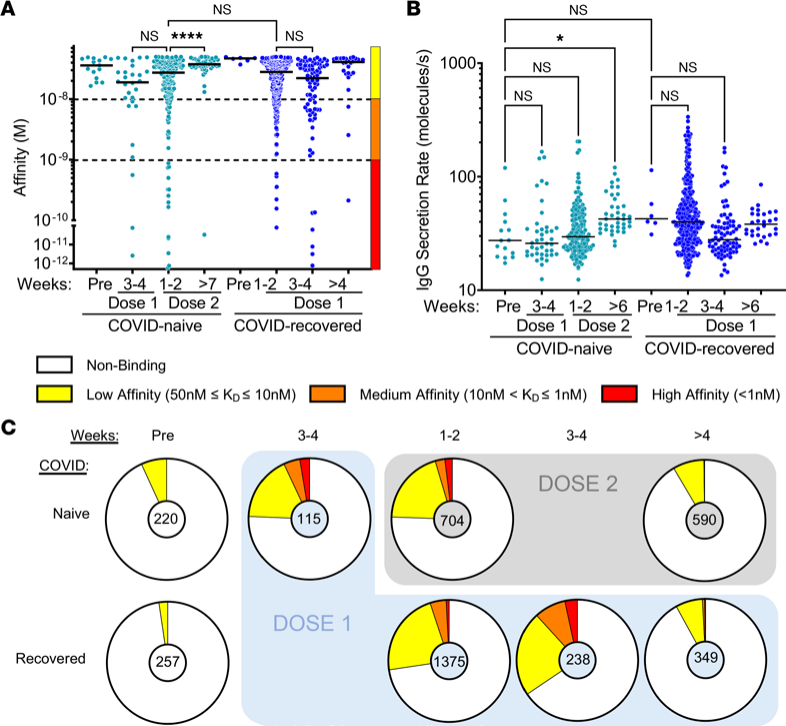
Anti–Hu-1 RBD IgG-SC responses following vaccination. (**A** and **B**) (**A**) Affinities for SARS-CoV-2 Hu-1 RBD and (**B**) secretion rates of a single IgG-SC (*n* = 762) from pooled vaccinee data, with naive and recovered individuals shown in light blue and dark blue, respectively. Each dot represents a value from a single cell. Medians are shown. NS, nonsignificant. *P < 0.05; ****P < 0.0001 using Kruskal-Wallis test with Dunn’s test for multiple comparisons. Affinities are grouped as low (yellow), medium (orange), and high (red) affinity, shown by the bar on the right and separated by the dotted lines. (**C**) Frequencies of RBD-specific IgG-SCs classified into low (yellow), medium (orange), high affinity (red), or no detectable binding (white), among total IgG-SCs (total numbers indicated in center).

Our 2 cohorts included a total of 29 individuals, with a median age of 47 years (ranging from 27 to 65 years). Clinical characteristics are presented in Supplemental Table 1 and anti–Hu-1 RBD titers in Supplemental Figure 2, A and B. To investigate the affinity and secretion rate of anti-RBD IgG-SCs, fresh blood samples were acquired in duplicate and analyzed on the same day, enabling the analysis of 10,000–20,000 single cells in total for each sample. We found that the frequency of IgG-SCs was from 0.01% to 2.5% of the mononuclear cell pool in the blood across all time points in the cohort. RBD-specific IgG-SCs could be identified in all samples, with high reproducibility in total and RBD-specific IgG-SC detection per replicate (exemplified in Supplemental Figure 2C). The 2 COVID-19–naive sampled before vaccination exhibited rare but detectable low-affinity (50 nM ≤ *K_D_* ≤ 10 nM, median of 34 nM) anti-RBD IgG–producing IgG-SCs (Figure 2A), even if their serum anti-RBD IgG titers were negative (Supplemental Figure 2A). IgG-SCs represented approximately 1% of total PBMCs, with approximately 6% RBD-binding among total IgG-SCs in both naive individuals. We also identified rare low-affinity anti-RBD ASCs in frozen prepandemic PBMC samples of 3 blood bank donors (Supplemental Figure 2D), suggesting that seasonal Betacoronavirus led to the generation of these cross-reactive low-affinity IgG-SCs to SARS-CoV-2 Hu-1 RBD. The 2 COVID-19–recovered individuals sampled before vaccination also exhibited only low-affinity (median of 47 nM) anti-RBD IgG–producing IgG-SCs, with IgG-SCs representing approximately 1% of total PBMCs and with 2% RBD-binding among total IgG-SCs (Figure 2A). Based on these data, we conclude that previous COVID-19 infection does not result in high-affinity IgG-SCs circulating in the blood after 6 months, and rather leaves no detectable RBD-specific IgG-SC signature that can be distinguished from previously uninfected individuals.

BNT162b2 vaccination induced an increase in anti-RBD IgG titers (Supplemental Figure 2, A and B) and in IgG-SC numbers in COVID-19–naive individuals readily detectable 3–4 weeks after the first dose, representing on average 24% of all IgG-SCs, with low (50 nM ≤ *K_D_* ≤ 10 nM), medium (10 nM < *K_D_* ≤ 1 nM), and high-affinity (*K_D_* < 1 nM) anti-RBD IgG-SCs (pooled data in Figure 2A; for individual data refer to Supplemental Figures 3 and 4). Rare IgG-SCs displayed very high estimated affinity (*K_D_* below 10^–10^ M) that were mathematically extrapolated from values outside the boundaries of the reference curve presented in Figure 1D, and led to a median affinity of 18 nM for anti-RBD IgG-SCs in that group. One to 2 weeks after receiving the second dose (booster, performed 27–29 days after the first dose), COVID-19–naive individuals had anti-RBD IgG-SCs (24% among total IgG-SCs) that displayed a similar range of affinities compared to 3–4 weeks after the first dose, but with a predominance of low-affinity IgG-SCs, leading to a median affinity of 28 nM (Figure 2A). Overall, more than 7 weeks after the second dose (53–71 days), anti-RBD IgG-SCs (~10% among IgG-SCs) were mainly low-affinity IgG-SCs, leading to a median affinity of 38 nM. This median affinity was comparable to nonvaccinated COVID-19–naive individuals, showing that RBD-specific ASCs generated after the boost rapidly disappeared from the circulation.

In the group of COVID-19–recovered individuals, BNT162b2 vaccination induced a large increase in anti-RBD IgG-SC numbers also within 1–2 weeks after the first vaccine dose, representing on average 27% of all IgG-SCs, with a large predominance of low-affinity over medium-affinity anti-RBD IgG-SCs, and only rare high-affinity anti-RBD IgG-SCs, resulting in a median affinity of 28 nM. No significant difference could be observed between vaccinated mild- and severe-COVID-19–recovered patients (Supplemental Figure 5A). Three to 4 weeks after the first dose, the distribution of affinities did not change significantly, with a median affinity of 22 nM (Figure 2A), and maintenance of a high proportion (34%) of anti-RBD IgG-SCs among IgG-SCs. More than 4 weeks after the first dose (31–63 days), low-affinity IgG-SCs represented almost all anti-RBD IgG-SCs (~10% among IgG-SCs) with rare exceptions, leading to a median affinity of 35 nM, which was similar to prevaccination values of these COVID-19–recovered individuals.

As exemplified in Figure 1E, in parallel with measuring affinity of the secreted IgG for SARS-CoV-2 RBD, DropMap allows measurement of the secretion rate of IgG within each droplet. Circulating anti-RBD IgG-SCs from all individuals in this cohort displayed a wide range of IgG secretion (12–320 IgG/s), with a global median value of approximately 36 IgG/s (Figure 2B). A significant increase (1.5-fold) in IgG secretion rate was detected more than 6 weeks after the second dose in COVID-19–naive individuals compared with prevaccination. Noticeably, this increase reached the levels found in COVID-19–recovered individuals (Figure 2B). No correlation was found, however, between affinity for RBD and IgG secretion rate using either the pooled data from all individuals or pooled data from subgroups of individuals (Supplemental Figure 5, B–K).

In terms of range of affinities and their distribution, the anti-RBD IgG-SC response appeared very similar between COVID-19–naive and COVID-19–recovered within 1 to 2 weeks after receiving the second and the first vaccine dose, respectively, with both cohorts displaying similar proportions between low-, medium-, and high-affinity IgG-SCs. Both groups demonstrated over the following weeks a rapid disappearance of high- and medium-affinity IgG-SCs in circulation, while keeping a similar fraction of low-affinity RBD-specific IgG-SCs among circulating IgG-SCs (Figure 2C).

Thus, in both cohorts, the recall response mobilizing RBD-specific B cells generated a few weeks after mRNA vaccination in COVID-19–naive or in COVID-19–recovered individuals (1 year after infection) elicits a burst of low-affinity anti-RBD IgG-SCs that rapidly wanes in the circulating blood.

### Anti–Omicron BA.1 RBD IgG-SC populations following vaccination in naive or recovered individuals.

**Figure 3:**
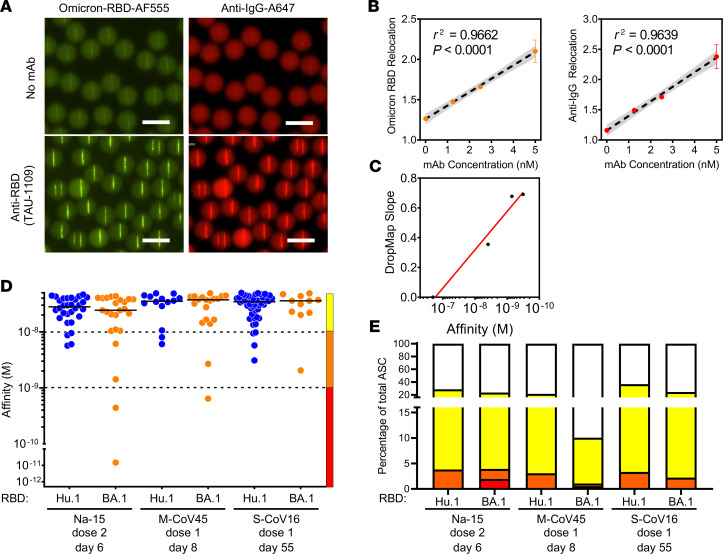
DropMap for the detection of anti-BA.1 IgG–secreting cells. (**A**) Example of droplets containing no mAb (top) or 5 nM TAU-1109 mAb (bottom), showing fluorescence relocation of Omicron BA.1 RBD–Alexa Fluor 555 (yellow, left) and anti–IgG F(ab′)_2_–Alexa Fluor 647 (red, right). Scale bars: 50 μm. (**B**) In-droplet mAb (TAU-1109) measurement of fluorescence relocation at 0, 1.25, 2.5, and 5 nM in droplet concentration performed in triplicate. Each dot represents the mean of a triplicate measure. Linear regression is represented, with 95% confidence intervals in gray, *n* = 3. (**C**) Omicron affinity reference curve calibrated with anti-RBD mAbs with known *K_D_* for BA.1 RBD and DropMap slope determined from method exemplified in **B**. (**D**) Affinities for SARS-CoV-2 RBD of a single IgG-SC (*n* = 181) from naive and recovered individuals using either Hu-1 (blue) or BA.1 (orange) RBD. Each dot represents a value from a single cell. Medians are shown. (**E**) Frequencies of Hu-1–specific (top) or BA.1-specific (bottom) IgG-SCs classified into low (yellow), medium (orange), high affinity (red), or no detectable binding (white), among total IgG-SCs, with total numbers indicated in center.

The Omicron BA.1 variant of SARS-CoV-2 appeared in France in early December, 2021, and we considered whether we could detect anti–BA.1 RBD cross-binding IgG-SCs in the samples collected before its emergence. Using fluorescently labeled BA.1 RBD, we generated a droplet bioassay (Figure 3A) and reference curve using BA.1 RBD cross-binding mAbs (Figure 3, B and C). Frozen samples of 1 vaccinated COVID-19–naive individual (Na-15; 41 y/o female; 6 days after second dose), 1 vaccinated mild-COVID-19–recovered individual (M-CoV-45; 41 y/o female; 8 days after first dose), and 1 vaccinated severe-COVID-19–recovered (S-CoV16; 61 y/o male; 55 days after first dose) individual were assayed in duplicate and in parallel the same day for measurement of affinity of their IgG-SCs for Hu-1 and BA.1 RBD. The distribution and affinity ranges of anti–Hu-1 RBD IgG-SCs in these thawed samples (Figure 3D) were similar to those detected in the fresh samples (panels Na-15, M-CoV-45, and S-CoV-16 in Supplemental Figures 3 and 4). Interestingly, anti–BA.1 RBD IgG-SCs could be detected in all 3 samples collected before the appearance of this SARS-CoV-2 variant, with 20%–50% fewer BA.1 RBD IgG-SCs compared with Hu-1 RBD IgG-SCs (Figure 3, D and E). Medium- and high-affinity anti–BA.1 RBD IgG-SCs were detected, with similar median affinities in each individual for BA.1 and Hu-1 RBD, and also between these 3 individuals. As in our analyses of anti–Hu-1 RBD IgG-SCs, low-affinity anti–BA.1 RBD IgG-SCs largely predominated in circulation over high-affinity IgG-SCs (Figure 3E). We conclude that approximately 50%–80% of the Hu-1 RBD IgG-SCs elicited by booster vaccination were cross-reactive with the Omicron BA.1 VOC.

## Discussion

This study demonstrates the power of the droplet-based microfluidic approach (DropMap) (22, 28) for the rapid follow-up of the human immune response to a pathogen, with results obtained within hours of sampling. Herein we provide a broad “affinity repertoire” characterization of circulating IgG-SCs over several time points following SARS-CoV-2 mRNA vaccination for the original Hu-1 SARS-CoV-2 RBD. It describes similar expansion and contraction of the circulating RBD-specific plasmablast pool following mRNA-based vaccination for prime/boosted COVID-19–naive and boosted COVID-19–recovered individuals, reaching high proportions (up to a third) of the total circulating plasmablast pool, in agreement with flow cytometry analyses (9, 30, 31) and proportions of RBD-specific antibodies among circulating antibodies (15).

We make the unanticipated observation that the majority of RBD-specific IgG-SCs were of lower affinity both in COVID-19–naive and –recovered individuals. The Ellebedy lab also reported that a large majority of plasmablasts producing antibodies with lower affinities — median of 80 nM — were also found in individuals who had received 2 doses of the BNT162b2 vaccine 6 months earlier (20). Unexpectedly, we identified in the prevaccination samples of 2 COVID-19–naive donors with no evidence of previous SARS-CoV-2 exposure, as well as in 3 prepandemic blood bank samples, rare IgG-SCs with low affinity for Hu-1 RBD. As Betacoronaviruses (OC43, HKU1) shared some, albeit limited, degree of homology with the Hu-1 RBD (32), this suggests, as previously described in the MBC compartment (7), a cross-reactivity against conserved epitopes. Alternatively, the detection of IgG-SCs with low affinity for Hu-1 RBD may also reflect intrinsic polyreactivity in the plasma cell compartment (33, 34).

In vaccinated COVID-19–naive individuals, the predominance of lower-affinity RBD-specific clones in circulation strongly suggest that they derive from extrafollicular maturation of activated B cells into plasmablasts harboring few mutations in IgVH, as described in patients with severe SARS-CoV-2 infection (35). By contrast, MBCs drive a recall response after antigenic rechallenge by differentiating into new ASCs that display the diverse array of high-affinity antibodies contained in the MBC repertoire, making the dominance of low-affinity clones quite unexpected. We have previously reported that some RBD-specific ASCs from COVID-19–recovered patients expressed enough B cell receptor (BCR) on the surface to be sorted 7 days after the boost (7). These clones displayed highly mutated V_H_ sequences corresponding to the recruitment of MBCs. The results obtained using DropMap suggest that while these high-affinity clones are mobilized, other clones with low affinity were also engaged in the response. Indeed, minor fractions of high-affinity (*K_D_* < 1 nM) anti-RBD IgG-SCs in both COVID-19–naive and –recovered individuals were detected with affinities reaching picomolar values.

The regular and frequent emergence of SARS-CoV-2 variants with mutations in the ACE2 RBD has complicated monitoring of the vaccination response against COVID-19. In this context, here we provide the first reported affinities to our knowledge of IgG-SCs against the Omicron BA.1 variant RBD. The median affinities between Hu-1 and BA.1 RBD–specific IgG-SCs were similar for the 3 samples we analyzed, but with a consistently lower frequency of BA.1-specific compared with Hu-1-specific IgG-SCs. These findings are largely consistent with existing analyses of the MBC compartment (16, 18, 19) and from neutralizing plasma titers (10). Numerous studies have found the prevalence of Hu-1 RBD–specific MBCs among the total MBC population to range between 0.01% and 10% (4, 5, 16, 29, 36–38), markedly lower than the prevalence of Hu-1 RBD–specific IgG-SCs among the total IgG-SC population (9, 31). Our results thus further support the notion that the reduced neutralization capacity of vaccinee plasma toward BA.1 SARS-CoV-2 is largely due to a drop in the number of antibodies recognizing the BA.1 RBD, as opposed to a global shift in affinity, within the context of RBD-specific clones.

The DropMap technique also allows for precise quantification of single-cell antibody secretion. The amount of Ig secreted by single IgG-SCs remains poorly characterized, with most studies assessing only global Ig concentrations or titers. Previous attempts to quantify IgG secretion by human IgG-SCs were performed only on in vitro–derived B cells and have reported a broad range of estimated secretion rates, including approximately 450,000 IgG/s (~1 ng/cell/day) (39) down to approximately 10 IgG/s (~20 fg/cell/day) (40). We have recently reported that ex vivo IgG-SCs from healthy individuals had IgG secretion rates between approximately 5 and 500 IgG/s in the blood, bone marrow, and spleen (28), which are consistent with the secretion rates we describe here for vaccine-induced IgG-SCs. Our data show that COVID-19–recovered individuals display a higher basal IgG secretion level in anti–Hu-1 RBD IgG-SCs compared with COVID-19–naive individuals. This heightened level of secretion was reached in COVID-19–naive individuals several weeks after the second dose of BNT162b2. More investigation would be valuable to further establish physiological secretion rates of IgG-SCs and what conditions either attenuate or potentiate Ig secretion over time, and to guide alternative vaccine design.

This study is the second application of DropMap to monitor human IgG-SCs, with several consistent findings regarding IgG-SC physiology between 2 different antigens (a human glycoprotein [28] versus a viral surface protein) and models driving IgG-SC generation (autoimmunity versus immunization). DropMap has also been used in other aspects of immune monitoring, including cytokine secretion by T cells from healthy or sepsis patients (26) and cytokine secretion and cytotoxicity from single human NK cells (27, 41). Despite the advantages afforded by droplet microfluidic techniques, it should be noted that such analyses require precise fabrication of imaging apparatuses and microfluidics consumables, which are currently not commercially available. Nonetheless, the affinity repertoire is a valuable component of the B cell responses to evaluate. Combining DropMap with other single-cell technologies, such as BCR sequencing and single-cell transcriptomics to correlate phenotype with genotype, and liquid chromatography–tandem mass spectrometry proteomics will ultimately allow for a more complete understanding of the key aspects in antibody-mediated immunity (reviewed in ref. 21).

This study provides direct ex vivo, sorting-free analyses to assess the prevalence and quality of antigen-specific cells among circulating IgG-SCs following human vaccination. Application of similar DropMap screenings following vaccination could be valuable to determine whether the proportions of specific IgG-SCs we observed is consistent between vaccine designs (adenovirus-, protein-, or mRNA-based), different types of pathogens (bacteria vs. viruses), and different antigens and doses. Together, these endeavors highlight the functional diversity of antibodies generated following immunization and offer demonstration of efficient, high-throughput methods for characterizing B cell responses.

## Methods

### Cloning, expression, and purification of SARS-CoV-2 RBD

The codon-optimized sequence encoding the SARS-CoV-2 RBD was downloaded from the NCBI database, synthesized by Syntezza, and cloned into the pcDNA3.1 mammalian expression vector. The construct contained an N-terminal signal peptide (MKAPAVLAPGILVLLFTLVQRSNG) and 2 C-terminal tags: a hexahistidine tag (His-tag) for downstream protein purification and a site-specific biotinylation tag (GLNDIFEAQKIEWHE, AviTag). The RBD-containing vector was used to transiently transfect Expi293F cells (Thermo Fisher Scientific) using the ExpiFectamine 293 Transfection Kit (Thermo Fisher Scientific) or FectoPRO (Polyplus). Seven days after transfection, the cell supernatant was collected, filtered (0.22 μm), and incubated with Ni^2+^-NTA agarose beads (Cytiva Life Sciences) for 2 hours at room temperature. The protein was eluted with 200 mM imidazole, buffer-exchanged to PBS, and aliquoted and stored at –80°C.

### Antibody expression and purification

The human mAbs used in this study (TAU-1109, TAU-2220, TAU-1115, TAU-2230, TAU-1145, TAU-2303, TAU-2189, TAU-2310, and mGO53) were isolated, cloned, and expressed as previously described (29, 42). Briefly, cloned antibody vectors for IgG1 heavy chain (H) and κ or λ light chains were cotransfected at a ratio of 1:3 (H/κ or H/λ) into Expi293F cells using the ExpiFectamine 293 Transfection Kit. Seven days after transfection, the cell supernatant was collected, filtered (0.22 μm), and incubated with protein A–coated agarose beads (GE Life Sciences) for 2 hours at room temperature. The antibodies were eluted using 50 mM sodium phosphate (pH 3.0) and the pH was immediately adjusted using 1 M Tris-HCl (pH 8.0). Following buffer exchange to PBS, the reactivity of each mAb was confirmed by ELISA before it was aliquoted and stored at –80°C.

### Protein labeling

NHS-ester dyes used included AF488 (FP-M17231, FluoProbes) and AF555 (FP-U90663, FluoProbes). The Hu-1 RBD was conjugated with AF488 and the BA.1 RBD with AF555 according to the manufacturer’s recommendations.

### Sample preparation

PBMCs were isolated from venous blood samples via standard density gradient centrifugation (Ficoll) and used fresh or after cryopreservation at –150°C in fetal bovine serum with 20% DMSO. PBMCs were enriched in ASCs by negative selection using anti-CD3 microbeads (Miltenyi Biotec) following the manufacturer’s protocol.

### Aqueous phase I: preparation of cells for droplet compartmentalization

Cell suspensions were centrifuged (300*g*, 5 minutes) and resuspended twice using MACS buffer (PBS pH 7.2, 0.2% bovine serum albumin, and 2 mM EDTA). After each resuspension, cells were filtered through a 40-μm cell strainer to eliminate aggregates. Cells were then spun (300*g*, 5 minutes) and resuspended in DropMap buffer (RPMI without phenol red supplemented with 0.1% Pluronic F68, 25 mM HEPES pH 7.4, 5% KnockOut serum replacement [Thermo Fisher Scientific], and 0.5% human serum albumin [Sigma-Aldrich]). Cell density was adjusted to achieve a mean number of cells per droplet of approximately 0.3. For calibration curves, monoclonal IgG antibodies were diluted in DropMap buffer.

### Aqueous phase II: preparation of beads and bioassay reagents

Paramagnetic nanoparticles (Strep Plus 300 nm, Ademtech) were washed with Dulbecco’s PBS with calcium and magnesium (DPBS++, Thermo Fisher Scientific). Nanoparticles were resuspended in DPBS++ containing 1 μM CaptureSelect biotin-labeled anti–human κ light chain (Igκ; 7103272100, Thermo Fisher Scientific) for Hu-1 RBD assays or CaptureSelect anti–human constant heavy chain 1 (IgG-CH1; 7103202100, Thermo Fisher Scientific) for assays shown in Figure 3, and incubated 20 minutes at room temperature. After another wash with DPBS++, nanoparticles were resuspended in 5% pluronic F127 solution (Thermo Fisher Scientific) and incubated 20 minutes at room temperature. The nanoparticles were washed again and resuspended in DropMap buffer containing fluorescent reporter proteins for a final concentration of 1.25 mg/mL beads. Reporter proteins included Alexa Fluor 647–labeled F(ab′)_2_ fragment of a rabbit anti–human IgG Fc-specific antibody (309-606-008, Jackson ImmunoResearch) used at 75 nM final in-droplet concentration; fluorophore-conjugated RBD(s) were used at 30 nM final in-droplet concentration.

### Droplet production and collection

Droplets were generated using hydrodynamic flow focusing on a microfluidic chip, as described previously (22). The wafer master of the SU-8 photoresist layer (MicroChem) approximately 40 μm in thickness was manufactured using soft lithography (43) and microfluidic chips were fabricated using soft lithography in polydimethylsiloxane (PDMS; Sylgard) (22). The continuous phase comprised 2% (wt/wt) 008 Fluorosurfactant (RAN Biotechnologies) in Novec HFE7500 fluorinated oil (3M). Aqueous phases I and II were co-flowed and partitioned into droplets. The flow rate of aqueous phases I and II was 70 μL/h, with an oil flow rate of 600 μL/h to achieve monodispersed droplets of approximately 40 pL volume. Newly generated droplets were directly injected into the DropMap 2D chamber system (22) and mounted on a widefield fluorescence microscope (Ti2-Eclipse, Nikon). The emulsion was exposed to a magnetic field, forcing the nanoparticles inside each droplet to form an elongated aggregate termed the “beadline.”

### Data acquisition

Images were acquired using a Nikon inverted microscope with a motorized stage (Ti2-Eclipse). Excitation light was provided by a light-emitting diode (LED) source (AURA III light source, Lumencor Inc.). Fluorescence for the specific channels was recorded using appropriate band-pass filters and camera settings (Orca Flash 4.0, Hamamatsu) at room temperature and ambient oxygen concentration. Images were acquired using a 10× objective (NA 0.45). An array of 9 × 9 images was acquired for each replicate, every 7.5 minutes, in all channels over 37.5 min (6 measurements total). A 9 × 9 image array describes a matrix of 81 individual 10× objective image acquisitions that are stitched together to form a single image with a height and width of 9 × (single image height/width). Each chamber (chip) contains droplets corresponding to a single sample, with the experiment repeated in duplicate. Duplicates were systematically acquired for every sample, with each replicate being the filling of the DropMap 2D chamber with a novel droplet population acquired over time on a 9 × 9 image array.

### Image analysis and calculations

Images were analyzed using a custom-made MATLAB script (MathWorks) to identify each droplet and its beadline. In each fluorescence channel and for each droplet, the pixel intensities of the beadline and the mean pixel intensities except the beadline (background fluorescence) were extracted. Droplet fluorescence relocation at each time point was calculated by dividing the intensity of the beadline by that of the background. Data were exported to Excel (Microsoft) and sorted for droplets showing an increase in relocation of the anti-IgG reporter fluorescence (Alexa Fluor 647) over time and above a threshold of Alexa Fluor 647 relocation greater than 1.3. Sorted droplets were visually assessed for the presence of a single cell within the droplet, no droplet movement between image acquisitions, the absence of fluorescent particles other than the beadline (e.g., fluorescent protein aggregates, cell debris), and undesired aggregation of fluorescent reporters on the cell surface inside the droplet. IgG secretion rate and dissociation constant (*K_D_*) were estimated as described previously (22).

#### Estimation of K_D_ for RBD within each droplet.

A reference curve was obtained by defining relocation values for anti-IgG (Alexa Fluor 647) and RBD with a panel of 7 anti-RBD mAbs with a 1 log *K_D_* range, determined using bio-layer interferometry (ForteBio). Droplet populations were generated with different concentrations of each mAb or buffer and the DropMap bioassay. A curve was defined by plotting the relocation from the anti-IgG (Alexa Fluor 647) against relocation of RBD, and the slope of the resulting line was calculated and termed “DropMap slope.” The reference curve was defined by the linear relationship between *K_D_* for RBD as defined by bio-layer interferometry and the DropMap slope of each mAb.

### Affinity determination of mAbs

Bio-layer interferometry measurements were performed using anti–human IgG sensors in an OctetHTX system (ForteBio). Biosensors were equilibrated in assay buffer (PBS with 0.05% Tween 20 [Xantec, B PBST 10-500] diluted to 1× in sterile water + 0.1% BSA) for at least 10 minutes prior to measurement. Anti-RBD mAbs (10 μg/mL) diluted in assay buffer were immobilized on sensors (90 seconds), followed by a baseline in assay buffer (30 seconds), association with Hu-1 or Omicron BA.1 RBD in solution across 6 serial 2-fold dilutions (300 seconds), and dissociation (600 seconds) steps. Traces were preprocessed by reference sample subtraction, alignment to the baseline average, and Savitzky-Golay filtering. *K_D_* was determined using a 1:1 binding model with global fitting in HT Analysis 11.1 software (ForteBio).

### Data availability

Assay microscopy images and droplet detections with beadline/droplet intensity measurements are available upon request.

### Statistics

GraphPad Prism version 9.4.1 was used for all analyses. Data were not normalized or normalized prior to being analyzed. All data points present single-cell values unless otherwise stated. Each sample (1 patient at a designated time point) was measured in 2 independent experiments and data points were pooled between duplicate experiments. Samples were not pooled unless explicitly stated. Affinity and secretion data were determined to be neither normally distributed nor log-normally distributed according to the D’Agostino and Pearson, Anderson-Darling, and Kolmogorov-Smirnov tests. The Kruskal-Wallis test was used for comparing samples or groups of samples, with Dunn’s multiple-comparison test for hypothesis testing. Statistical significance was set to a *P* value of 0.05 or less.

### Study approval

This study was conducted in compliance with the Declaration of Helsinki principles and was approved by the Agence de la Biomédecine and the Institutional Review Board Comité de Protection des Personnes (CPP) Ile-de-France VI (number 40-20 HPS).

All patients provided written informed consent before the collection of samples. In total, 34 COVID-19–recovered patients from the original MEMO-COV-2 cohort were followed up to 12 months after infection and/or vaccination. Among them, 17 patients had severe COVID-19 (patients requiring oxygen, S-CoV) and 17 had a mild COVID-19 disease (mainly healthcare workers, M-CoV). An additional cohort of 9 patients who experienced mild COVID-19 during the first wave and were vaccinated at least 6 months after the infection were recruited. SARS-CoV-2 infection was defined as confirmed reverse transcriptase polymerase chain reaction (RT-PCR) on nasal swab or clinical presentation associated with typical aspect on CT scan and/or serological evidence. Twenty-five healthcare workers who had no history of COVID-19 and negative IgG anti-nucleocapsid (and/or anti-spike) were enrolled in the naive group (IRB 2018-A01610-55).

All vaccinated participants received the BNT162b2 mRNA vaccine. COVID-19–recovered patients received only 1 dose, in line with French guidelines. The first injection was realized in a mean of 309 days (±44.6 days SD) after the infection. Naive patients received 2 doses at a mean of 27.7 days (±1.8 days SD).

Prior to vaccination, samples were collected from COVID-19–recovered patients 12 months after symptoms onset (mean ± SD 329.1 ± 15: 8.8 days after disease onset for S-CoV, and 342.0 ± 8.6 days after disease onset for M-CoV). Samples at 12 months after disease onset were defined as “pre-boost.” For patients not sampled before vaccination (*n* = 9/34), the sample at 6 months was considered “pre-boost.” For naive patients, the “prime” time point was defined as the sampling between the 2 doses and was drawn at a mean of 20.2 ± 5.9 days after the first vaccine injection.

Samples were additionally collected shortly after the boost (mean ± SD: 10 ± 5.3 days for S-CoV, 23 ± 6.1 days for M-CoV, and 9 ± 4.0 days for naive), and 2 months after the boost (mean ± SD: 64.7 ± 15.3 days for S-CoV, 63.2 ± 11.9 days for M-CoV, and 63.3 ± 9.0 days for naive). Clinical and biological characteristics of these patients are summarized in Supplemental Table 1. Patients were recruited at the Henri Mondor University Hospital (AP-HP) between March and April 2021. The MEMO-COV-2 study (ClinicalTrials.gov NCT04402892) was approved by the ethical committee Ile-de-France VI (number 40-20 HPS), and was performed in accordance with French law. Written informed consent was obtained from all participants.

## Author contributions

M Broketa, NTF, M Mahévas, and PB conceptualized the study and developed the methodology. M Broketa and AS curated the data. M Broketa, NTF, M Mahévas, and PB formally analyzed and validated the data. M Broketa, AS, M Backovic, AM, IF, M Mor, PCH, AP, BI, and GC carried out the investigation. JB, NTF, M Mahévas, and PB acquired funding. AV, LL, M Michel, BG, SG, GM, and FAR provided resources. M Broketa, AP, GC, and JB contributed to code writing and software development. M Broketa generated the figures. FAR, JB, NTF, M Mahévas, and PB supervised the study. M Mahévas and PB provided project administration. M Broketa, NTF, M Mahévas, and PB wrote the original draft of the manuscript. All authors reviewed and edited the manuscript.

## Supplementary Material

Supplemental data

Supplemental table 1

## Version 1 05/30/2023

In-Press Preview

## Version 2 07/10/2023

Electronic publication

## References

[1] Yuan M (2020). Structural basis of a shared antibody response to SARS-CoV-2. Science.

[2] Cho A (2021). Anti-SARS-CoV-2 receptor-binding domain antibody evolution after mRNA vaccination. Nature.

[3] Gaebler C (2021). Evolution of antibody immunity to SARS-CoV-2. Nature.

[4] Goel RR (2021). Distinct antibody and memory B cell responses in SARS-CoV-2 naïve and recovered individuals following mRNA vaccination. Sci Immunol.

[5] Wec AZ (2020). Broad neutralization of SARS-related viruses by human monoclonal antibodies. Science.

[6] Amanat F (2021). SARS-CoV-2 mRNA vaccination induces functionally diverse antibodies to NTD, RBD, and S2. Cell.

[7] Sokal A (2021). Maturation and persistence of the anti-SARS-CoV-2 memory B cell response. Cell.

[8] Levin EG (2021). Waning immune humoral response to BNT162b2 Covid-19 vaccine over 6 months. N Engl J Med.

[9] Turner JS (2021). SARS-CoV-2 mRNA vaccines induce persistent human germinal centre responses. Nature.

[10] Gupta SL (2022). Loss of Pfizer (BNT162b2) vaccine-induced antibody responses against the SARS-CoV-2 Omicron variant in adolescents and adults. J Virol.

[11] DeGrace MM (2022). Defining the risk of SARS-CoV-2 variants on immune protection. Nature.

[12] Laidlaw BJ, Ellebedy AH (2022). The germinal centre B cell response to SARS-CoV-2. Nat Rev Immunol.

[13] Tao K (2022). Susceptibility of SARS-CoV-2 Omicron variants to therapeutic monoclonal antibodies: systematic review and meta-analysis. Microbiol Spectr.

[14] Takashita E (2022). Efficacy of antibodies and antiviral drugs against Omicron BA.2.12.1, BA.4, and BA.5 subvariants. N Engl J Med.

[15] Wang Z (2021). Naturally enhanced neutralizing breadth against SARS-CoV-2 one year after infection. Nature.

[16] Muecksch F (2022). Increased memory B cell potency and breadth after a SARS-CoV-2 mRNA boost. Nature.

[17] Sokal A (2021). mRNA vaccination of naive and COVID-19-recovered individuals elicits potent memory B cells that recognize SARS-CoV-2 variants. Immunity.

[18] Kotaki R (2022). SARS-CoV-2 Omicron-neutralizing memory B cells are elicited by two doses of BNT162b2 mRNA vaccine. Sci Immunol.

[19] Sokal A (2022). Analysis of mRNA vaccination-elicited RBD-specific memory B cells reveals strong but incomplete immune escape of the SARS-CoV-2 Omicron variant. Immunity.

[20] Kim W (2022). Germinal centre-driven maturation of B cell response to mRNA vaccination. Nature.

[21] Broketa M, Bruhns P (2021). Single-cell technologies for the study of antibody-secreting cells. Front Immunol.

[22] Eyer K (2017). Single-cell deep phenotyping of IgG-secreting cells for high-resolution immune monitoring. Nat Biotechnol.

[23] Heo M (2020). Deep phenotypic characterization of immunization-induced antibacterial IgG repertoires in mice using a single-antibody bioassay. Commun Biol.

[24] Krautler NJ (2020). Quantitative and qualitative analysis of humoral immunity reveals continued and personalized evolution in chronic viral infection. Cell Rep.

[25] Bonaud A (2023). Sec22b is a critical and nonredundant regulator of plasma cell maintenance. Proc Natl Acad Sci U S A.

[26] Bounab Y (2020). Dynamic single-cell phenotyping of immune cells using the microfluidic platform DropMap. Nat Protoc.

[27] Subedi N (2021). An automated real-time microfluidic platform to probe single NK cell heterogeneity and cytotoxicity on-chip. Sci Rep.

[28] Canales-Herrerias P (2022). High-affinity autoreactive plasma cells disseminate through multiple organs in patients with immune thrombocytopenic purpura. J Clin Invest.

[29] Mor M (2021). Multi-clonal SARS-CoV-2 neutralization by antibodies isolated from severe COVID-19 convalescent donors. PLoS Pathog.

[31] Pape KA (2021). High-affinity memory B cells induced by SARS-CoV-2 infection produce more plasmablasts and atypical memory B cells than those primed by mRNA vaccines. Cell Rep.

[32] Hicks J (2021). Serologic cross-reactivity of SARS-CoV-2 with endemic and seasonal Betacoronaviruses. J Clin Immunol.

[33] Yurasov S (2005). Defective B cell tolerance checkpoints in systemic lupus erythematosus. J Exp Med.

[34] Scheid JF (2011). Differential regulation of self-reactivity discriminates between IgG^+^ human circulating memory B cells and bone marrow plasma cells. Proc Natl Acad Sci U S A.

[35] Woodruff MC (2020). Extrafollicular B cell responses correlate with neutralizing antibodies and morbidity in COVID-19. Nat Immunol.

[36] Robbiani DF (2020). Convergent antibody responses to SARS-CoV-2 in convalescent individuals. Nature.

[37] Rogers TF (2020). Isolation of potent SARS-CoV-2 neutralizing antibodies and protection from disease in a small animal model. Science.

[38] Dan JM (2021). Immunological memory to SARS-CoV-2 assessed for up to 8 months after infection. Science.

[39] Brinkmann V, Heusser CH (1993). T cell-dependent differentiation of human B cells into IgM, IgG, IgA, or IgE plasma cells: high rate of antibody production by IgE plasma cells, but limited clonal expansion of IgE precursors. Cell Immunol.

[40] Huggins J (2007). CpG DNA activation and plasma-cell differentiation of CD27- naive human B cells. Blood.

[41] Subedi N (2023). Single-cell profiling reveals functional heterogeneity and serial killing in human peripheral and ex vivo-generated CD34^+^ progenitor-derived natural killer cells. Adv Biol (Weinh).

[42] Wardemann H (2003). Predominant autoantibody production by early human B cell precursors. Science.

[43] Mazutis L (2013). Single-cell analysis and sorting using droplet-based microfluidics. Nat Protoc.

