# Supplementary material for: Qualitative monitoring of SARS-CoV-2 mRNA vaccination in humans using droplet microfluidics
Source: JCI Insight. 2023 Jul 10;8(13):e166602. doi: 10.1172/jci.insight.166602 (PMC10371332; doi:10.1172/jci.insight.166602)
Supplement: Supplemental data [file jciinsight-8-166602-s190.pdf]

A

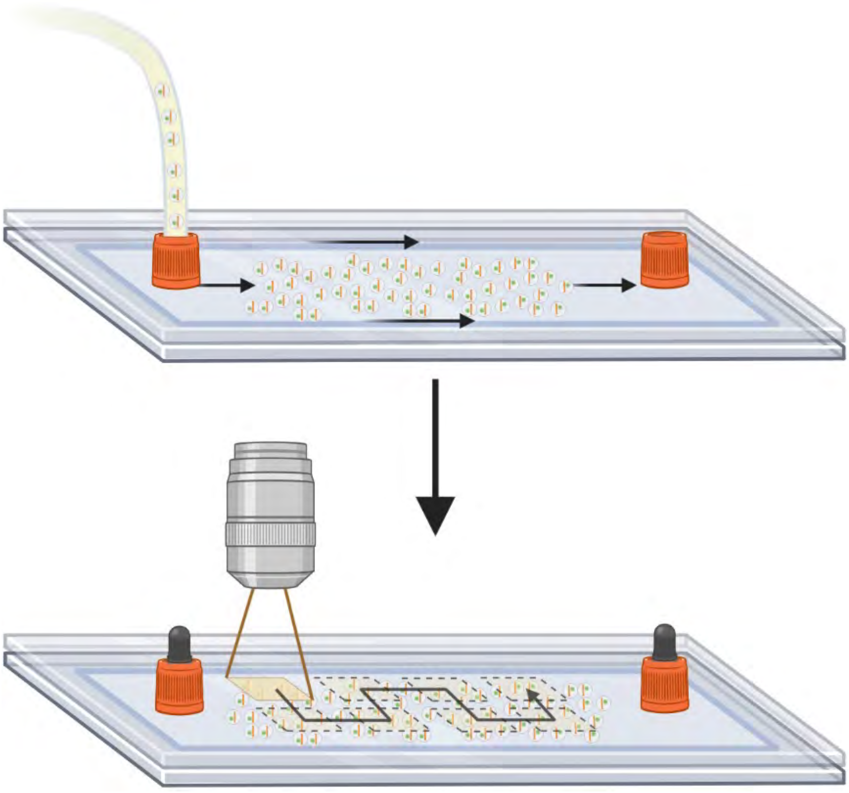

B

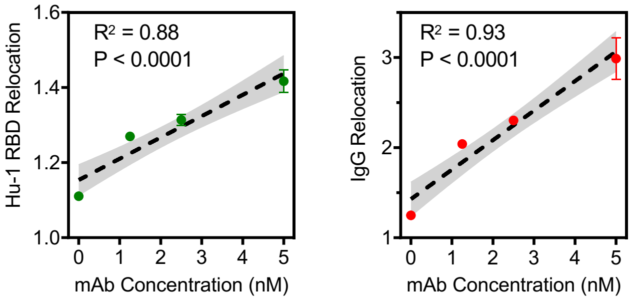

Supplementary Figure 1. (**A**) Schematic of the DropMap chamber (top) filling with droplets in an oil flow from the left inlet (red). Arrows indicate the direction of the flow. (Bottom) Schematic of the DropMap chamber with closed inlets (black caps) containing immobilized droplets that is visualized by time-lapse microscopy (objective and field of view depicted), generating a 9x9 matrix of 81 individual 10x-objective image acquisitions for each time point. (**B**) In-droplet anti-RBD mAb (mAb TAU-1109) measurement of fluorescence relocation at 0, 1.25, 2.5, and 5 nM in-droplet concentration, performed in triplicate. Each dot represents the mean of the triplicate measure. Linear regression is represented, with 95% confidence intervals in gray.

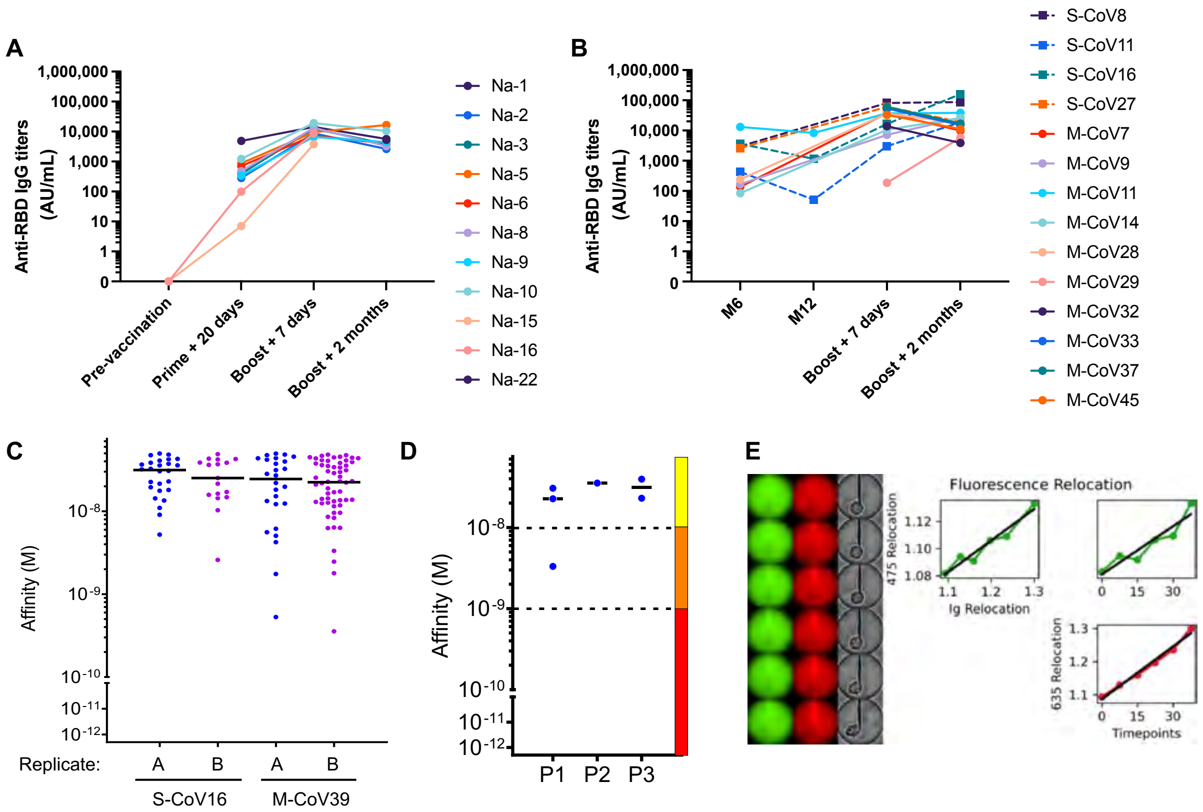

Supplementary Figure 2. (**A-B**) Serum anti-RBD IgG titers from (A) COVID-naive donors and (B) COVID-recovered patients at indicated timepoints. Each dot represents one patient and patient identification numbers are indicated. M6, 6-months after SARS-CoV-2 infection; M12, 12-months after SARS-CoV-2 infection. The pre-vaccination samples correspond to donors Na-15 and Na-16. (**C**) Reproducible affinity measurement between technical DropMap replicates. Affinities towards SARS-CoV-2 Hu-1 RBD of single IgG-SC from representative COVID-recovered individuals. First (A; blue) and second (B; violet) technical replicates are shown for each sample. Each dot represents a value from a single cell. Median values are represented by a horizontal bar. (**D**) Affinities towards SARS-CoV-2 Hu-1 RBD of single IgG-SC from 3 blood bank donors whose PBMCs were cryo-preserved in 2017 prior to SARS-CoV-2 emergence (P1, n= 221 IgG-SC analyzed; P2, n= 9 IgG-SC analyzed; P3, n=52 IgG-SC analyzed). Each dot represents a value from a single cell. P1 and P3 represent the aggregate of 3 replicate acquisitions and P2 a single acquisition. (**E**) Representative analysis report of one RBD-specific IgG-SC-containing droplet from a pre-pandemic donor. The image shows the evolution of an individual droplet from the first timepoint (t = 0 min) to the final timepoint (t = 37.5 min), with each component of the assay shown its detection channel, Hu-1 RBD-AF488 in green (475 channel), anti-human-IgG F(ab')2-AF647 shown in red (635 channel), and the cell and beadline shown in brightfield. The two right-hand graphs show relocation measurements (dividend of mean beadline fluorescence over mean droplet fluorescence, y-axis) in the RBD (top, green) and IgG (red, bottom) detection channels over time (x-axes). The left-hand graph shows antigen relocation (y-axis) against IgG relocation (x-axis), and represents the "DropMap slope".

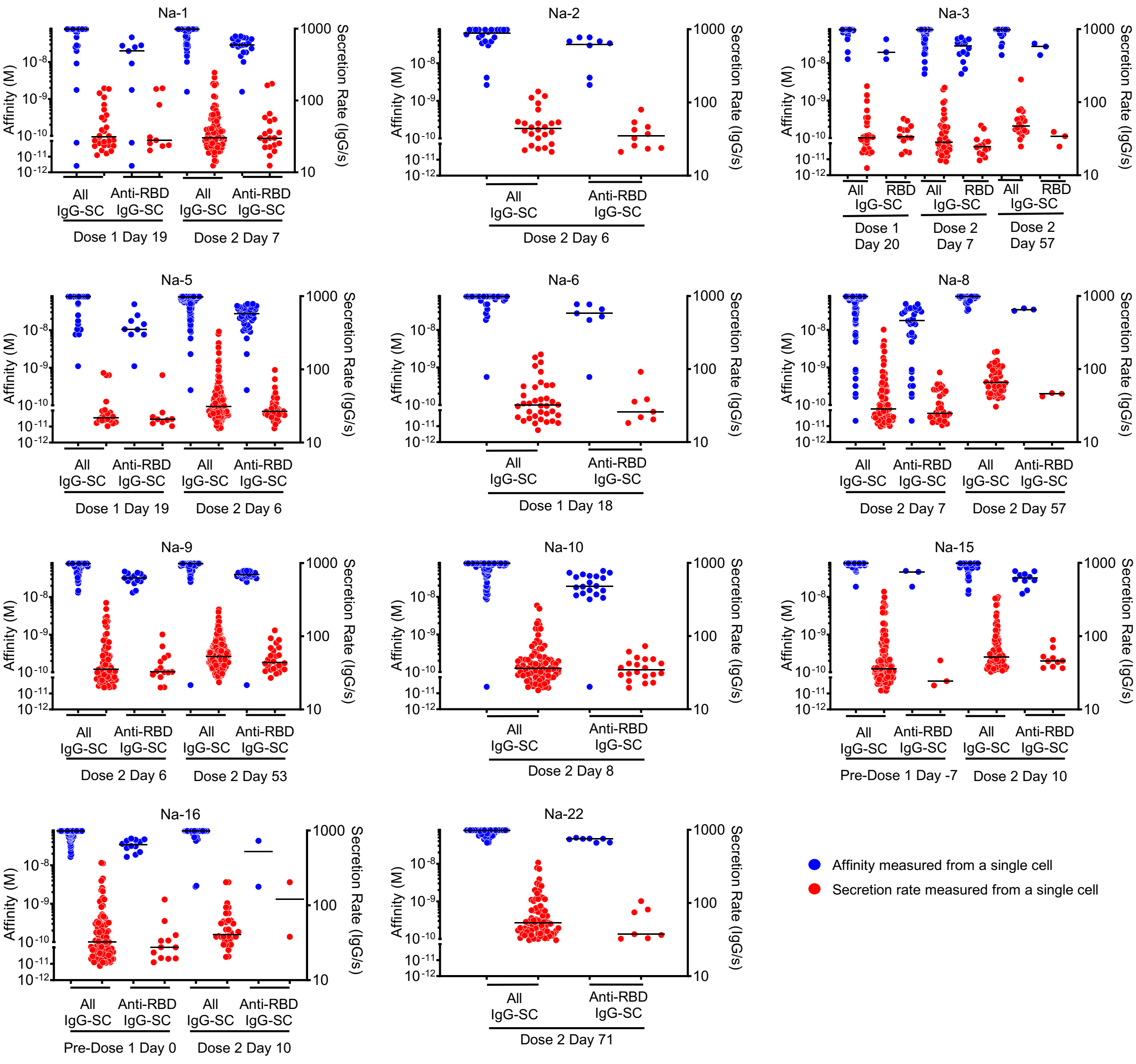

Supplementary Figure 3. Individual IgG-SC affinities and secretion rates obtained from COVID-naive individuals. Affinities (blue, left axis) and secretion rates (red, right axis) of single IgG-SCs from COVID-naive individuals and all timepoints measured (vaccine dose and days after the vaccine dose are indicated). "All IgG-SC" shows affinity and secretion data for all detected IgG-SC and "Anti-RBD IgG-SC" shows only affinity and secretion from IgG-SC with antibody affinity measured below $5x10^{-8}$ M for Hu-1 RBD. Each dot represents a value from a single cell; median values are represented by a horizontal bar.

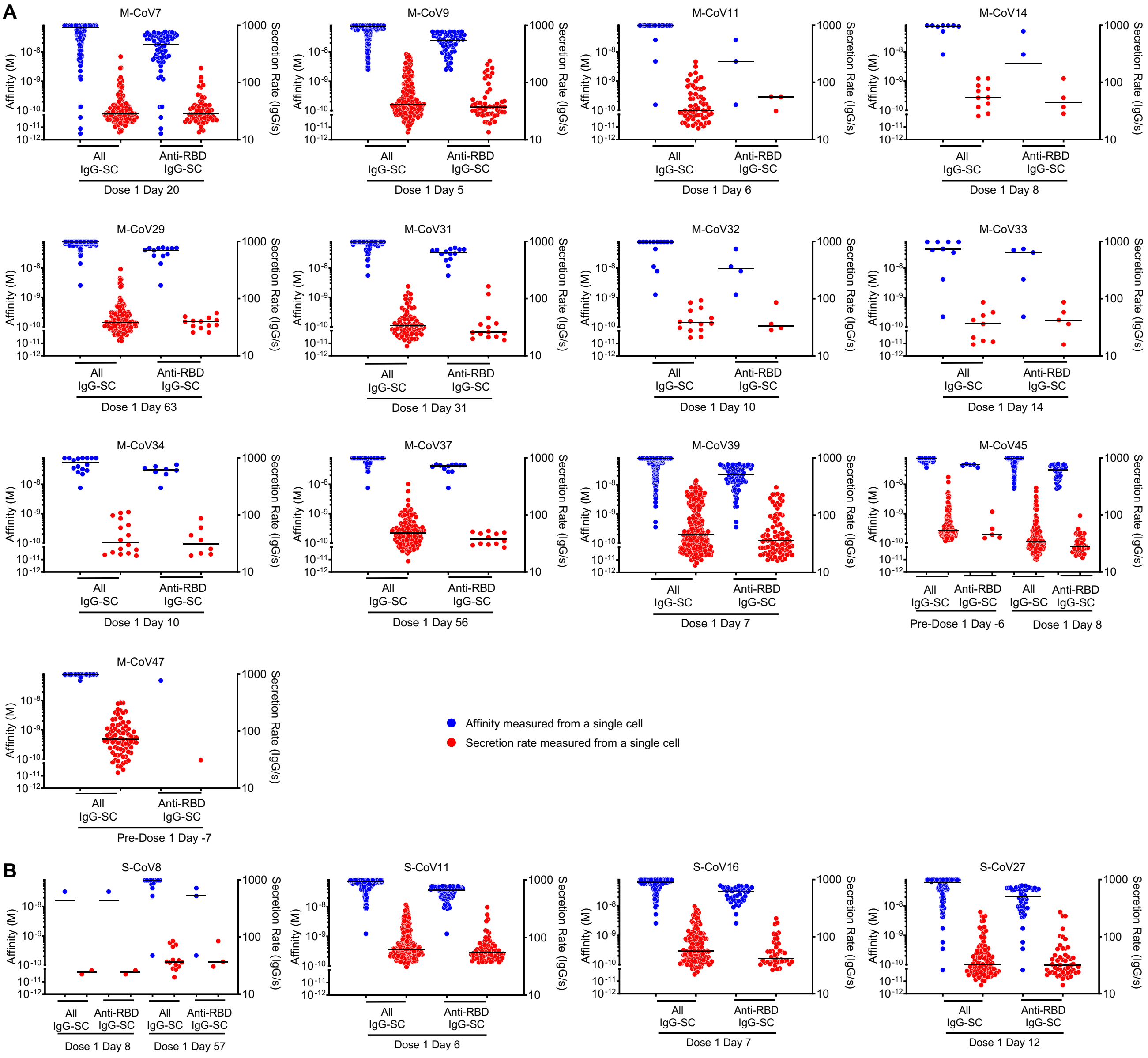

Supplementary Figure 4. Individual IgG-SC affinities and secretion rates obtained from COVID-recovered individuals. Affinities (blue, left axis) and secretion rates (red, right axis) of single IgG-SCs from (A) mild or (B) severe COVID-recovered individuals and all timepoints measured (vaccine dose and days after the vaccine dose are indicated). "All IgG-SC" shows affinity and secretion data for all detected IgG-SC and "Anti-RBD IgG-SC" shows only affinity and secretion from IgG-SC with antibody affinity measured below 5×10^{-8} M for Hu-1 RBD. Each dot represents a value from a single cell. Median values are represented by a horizontal bar.

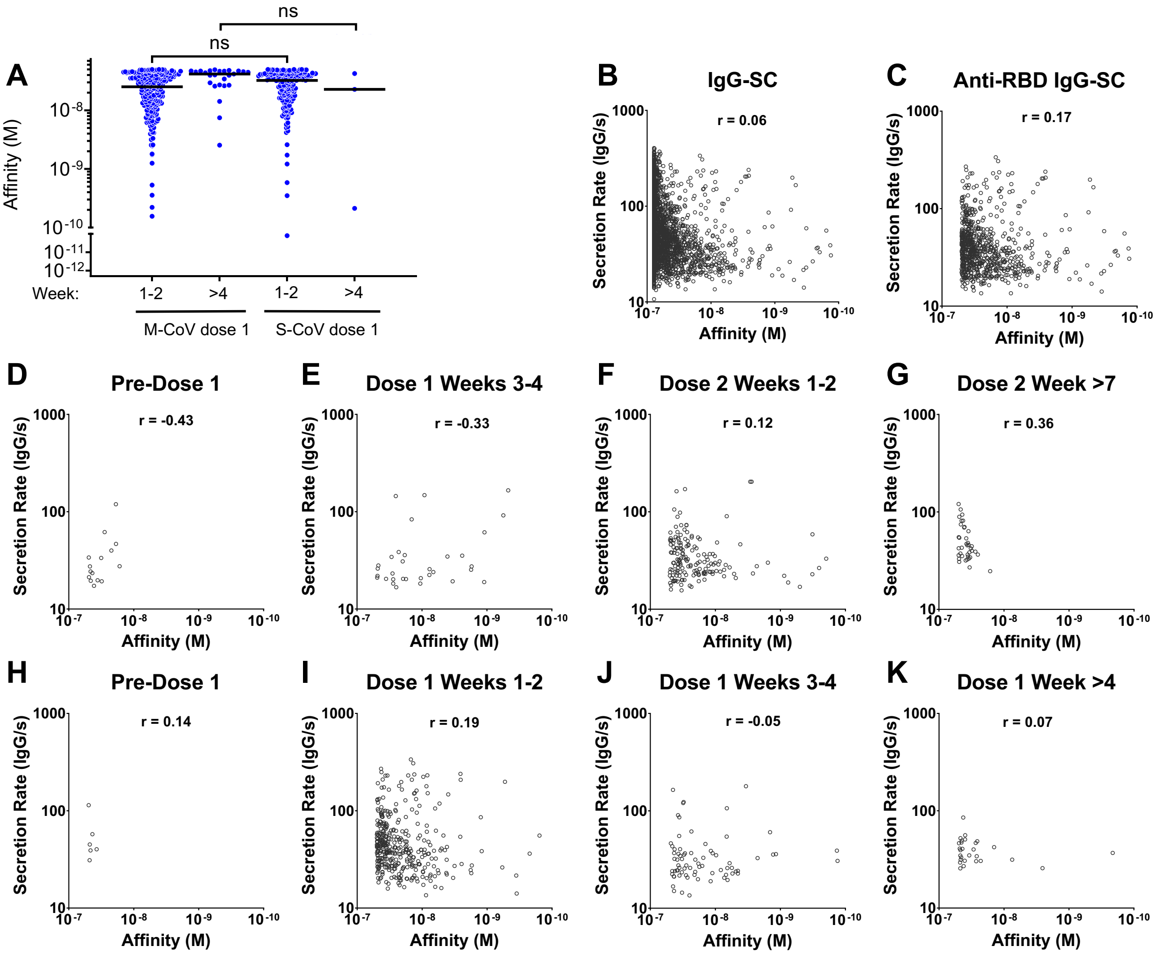

Supplementary Figure 5. (A) *Comparison of pooled data from M-CoV and S-CoV IgG-SC affinities for Hu-1 RBD*. Affinities towards SARS-CoV-2 Hu-1 RBD of single IgG-SC from pooled COVID-recovered vaccinee data, separated according to COVID severity. Each dot represents a value from a single cell. Median values are represented by a horizontal bar. Significance tested using Kolmogorov-Smirnov test. ns, not significant. (**B-K**) *Lack of correlation between IgG secretion rate and affinity for SARS-CoV-2 Hu-1 RBD.* Single cell IgG secretion rate by IgG-SC plotted against their affinity towards SARS-CoV-2 Hu-1 RBD from COVID naive (B-G) or recovered (H-K) individuals, with number of doses and time elapsed following immunization indicated. (B) All detected IgG-SC represented. (C-K) Plots restricted to IgG-SC with antibody affinity towards Hu-1 RBD of less than $5x10^{-8}$ M. r represents the correlation coefficient of Spearman's correlation analysis. Each dot represents a value from a single cell.
